# ASB7 promotes osteosarcoma lung metastasis through ubiquitin-mediated degradation of ATF2

**DOI:** 10.1038/s41421-026-00890-9

**Published:** 2026-05-05

**Authors:** Yezi Zou, Jianliang Zhong, Lanqing Huo, Jiali Chen, Xinhao Yu, Jingxuan Wang, Zhenxuan Chen, Lifeng Yin, Cuiling Zeng, Xia Zhang, Shan Han, Ruhua Zhang, Xing-Ding Zhang, Tiebang Kang, Liwen Zhou

**Affiliations:** 1 Sun Yat-sen University Cancer Center, State Key Laboratory of Oncology in South China, Guangdong Provincial Clinical Research Center for Cancer, Guangzhou, Guangdong, China; 2 Shenzhen Key Laboratory for Systems Medicine in Inflammatory Diseases, School of Medicine, Shenzhen Campus of Sun Yat-Sen University, Sun Yat-Sen University, Shenzhen, Guangdong, China; 3 Department of Musculoskeletal Oncology, The First Affiliated Hospital, Sun Yat-sen University, Guangzhou, Guangdong, China; 4 Department of Radiation Oncology, The Third Affiliated Hospital of Kunming Medical University, Tumor Hospital of Yunnan Province, Kunming, Yunnan, China

**Keywords:** Bone cancer, Metastasis, Proteolysis

## Abstract

Osteosarcoma is a highly heterogeneous and aggressive malignancy with a strong propensity for metastasis, highlighting the need to define its molecular drivers. Here, we report that ASB7 promotes tumor cell protrusion formation, invasion, migration, and lung metastasis. High expression of ASB7 is correlated with a poor prognosis. ASB7 forms an E3 ubiquitin ligase complex with CUL5 to ubiquitinate ATF2 at K383 and promote its proteasomal degradation. ATF2 reduction impairs HDAC6 recruitment to the ITGB2 promoter, thereby alleviating the transcriptional repression of ITGB2. Elevated ITGB2 expression subsequently promotes tumor lung metastasis. Our findings reveal that the ASB7-ATF2/HDAC6-ITGB2 axis regulates osteosarcoma metastasis and suggest potential treatment targets.

## Introduction

Osteosarcoma is a primary malignant bone tumor that predominantly affects children, adolescents, and elderly individuals (over 65 years of age)^1^. Characterized by somatic copy-number alterations and structural rearrangements, osteosarcoma has high metastatic potential, with lung metastases being most prevalent^2^. Despite the significant clinical challenge of treating metastatic osteosarcoma, recent advances in high-throughput genomic sequencing have substantially advanced our understanding of its pathogenesis, offering opportunities to overcome current therapeutic limitations^3–7^.

ASB7 serves as a critical substrate recognition component of the Cullin5-RING E3 ubiquitin ligase complex^8,9^. ASB7 maintains genome integrity by facilitating DDA3 degradation and negatively regulates H3K9me3 homeostasis through SUV39H1 degradation^10,11^. In addition, ASB7 coordinates cytoskeletal reorganization and participates in endoplasmic reticulum stress responses, underscoring its involvement in multiple essential biological processes^12,13^. Our previous work revealed that elevated ASB7 expression in tumor cells leads to reduced H3K9me3 levels, impaired homologous recombination, and genomic instability^11^. Moreover, TCGA pan-cancer data show frequent ASB7 genomic amplification across various cancers, with the highest frequency observed in sarcoma^11^, suggesting that ASB7 may play an important role in tumor progression.

In this study, we report that ASB7 is a key pro-metastatic factor. DepMap analysis revealed that both ASB7 expression and genomic amplification are elevated in osteosarcoma cell lines compared with other tumor types. Functionally, ASB7 overexpression promoted lung metastasis in vivo, and its high expression was found to be correlated with poor clinical outcomes. ASB7 forms an E3 ligase complex with CUL5 to ubiquitinate ATF2 at K383 and promote its proteasomal degradation. Reduced ATF2 impaired the recruitment of HDAC6 to the ITGB2 promoter, thereby alleviating transcriptional repression. Consequently, upregulated ITGB2 promoted cell protrusion formation, migration, invasion and lung metastasis.

## Results

### ASB7 promotes osteosarcoma cell invasion, migration, and lung metastasis

**Fig. 1:**
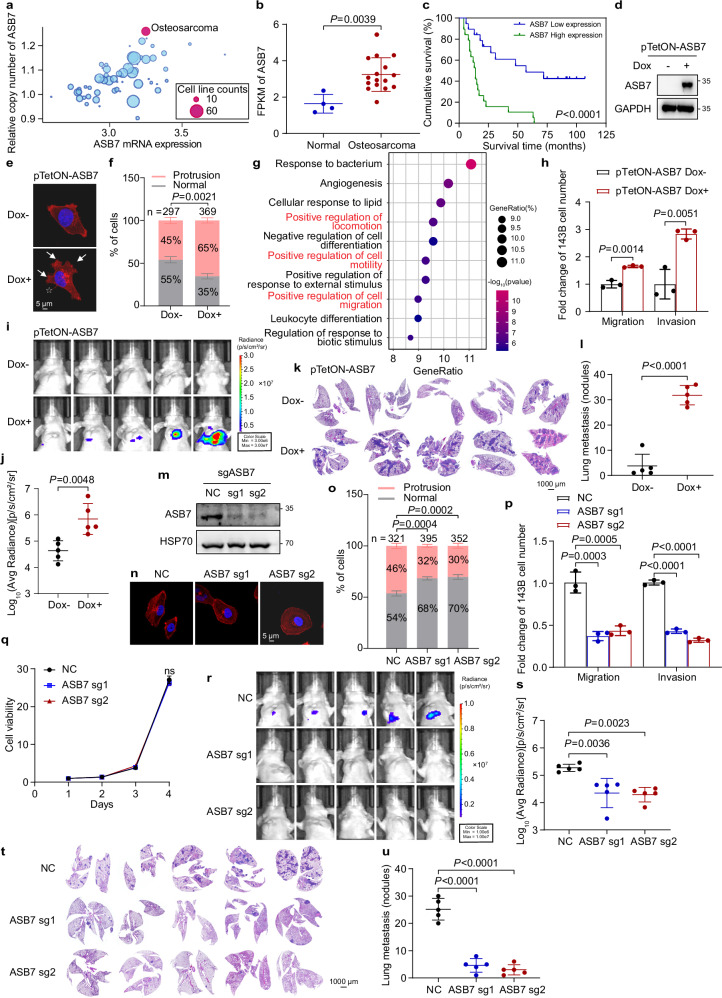
ASB7 drives osteosarcoma cell invasion, migration, and lung metastasis. **a** The relative copy number, mRNA expression of ASB7 and cell line count of each tumor type were plotted using 25Q2 DepMap cancer cell line data. **b** RNA-seq analysis of ASB7 mRNA levels in normal (*n* = 4) and osteosarcoma (*n* = 16) tissues. **c** Kaplan-Meier survival curves comparing osteosarcoma patients with low versus high ASB7 protein expression (low, *n* = 19; high, *n* = 19). The cutoff value for distinguishing the low- and high-expression groups was defined as the median IHC score obtained from the HALO system. The corresponding *P* value from the log-rank test is shown. **d**, **e**, **h** 143B cells stably expressing doxycycline-inducible ASB7 overexpression were treated with or without 0.5 μg/mL doxycycline for 24 h. After that, the cells were subjected to western blotting to verify the upregulation of ASB7 expression (**d**), immunofluorescence staining with Actin-Tracker (**e**), and migration and invasion assays (*n* = 3 independent experiments) (**h**). **f** The percentage of cells with protrusions was quantified in 143B cells overexpressing ASB7. Cells exhibiting finger-like filopodia (marked by asterisks) or fan-/wave-like lamellipodia (indicated by arrowheads), as shown in **e**, were scored as positive. **g** GO analysis of genes upregulated in 143B cells overexpressing ASB7. **i**–**l** Luciferase-expressing 143B cells with a doxycycline-inducible ASB7 overexpression system were injected into mouse tibiae. After that, the mice were fed either a control diet or a diet containing 0.065% doxycycline. Lung metastases were assessed by IVIS imaging (**i**) and quantitative bioluminescence analysis (**j**). Lung tissue sections were subjected to H&E staining (**k**), and metastatic foci were quantified (**l**). *n* = 5 mice per group. **m**–**q** 143B cells expressing sgRNAs of either non-target control or ASB7 were subjected to western blotting to detect the expression of ASB7 (**m**), immunofluorescence staining with Actin-Tracker (**n**), a quantification of cells with finger-like or fan-/wave-like protrusions (**o**), migratory and invasive assays (*n* = 3 independent experiments) (**p**), and an MTT assay (*n* = 3 independent experiments) (**q**). **r**–**u** Mice implanted with 143B cells expressing both luciferase and sgRNAs targeting either non-target control or ASB7 were analyzed by bioluminescence imaging, IVIS images are shown (**r**), and the data are plotted (**s**). Lung sections from these mice were analyzed by H&E staining (**t**), and the quantification of metastatic foci (**u**) is plotted. *n* = 5 mice per group. Data are shown as the mean ± S.D. *P* values were derived from unpaired two-tailed Student’s *t*-test for the data in **b**, **f**, **h**, **j**, **l**, **o**, **p**, **s**, **u** and from two-way ANOVA followed by Tukey’s multiple comparisons test for the data in **q**.

To investigate the role of ASB7 in tumor progression, we first analyzed DepMap cell line data and found that osteosarcoma cell lines exhibit both strong genomic amplification and high expression of ASB7 (Fig. 1a). Consistently, RNA-seq analysis revealed elevated ASB7 expression in osteosarcoma tissues (Fig. 1b). Notably, high ASB7 expression was associated with poor clinical outcomes, indicating that ASB7 is an important regulator of osteosarcoma progression (Fig. 1c).

To investigate the biological function of ASB7 in osteosarcoma, we established a doxycycline (Dox)-inducible ASB7 overexpression system in 143B cells (Fig. 1d). Immunofluorescence staining using Actin-Tracker revealed increased formation of actin-based protrusions, including lamellipodia and filopodia, upon ASB7 induction (Fig. 1e, f). Genes correlated with cell migration were upregulated in cells overexpressing ASB7 (Fig. 1g), suggesting that ASB7 plays a pro-migratory role. Indeed, functional assays demonstrated that induced ASB7 overexpression promoted the migration and invasion of osteosarcoma cells (Fig. 1h). Furthermore, mouse orthotopic xenograft experiments revealed that induced ASB7 overexpression increased lung metastases (Fig. 1i–l). In contrast, ASB7-knockout suppressed cell protrusion formation, migration, invasion and tumor metastasis (Fig. 1m–u). Collectively, these data reveal that ASB7 is an important regulator of osteosarcoma metastasis.

### ATF2 suppresses osteosarcoma cell invasion, migration, and lung metastasis

**Fig. 2:**
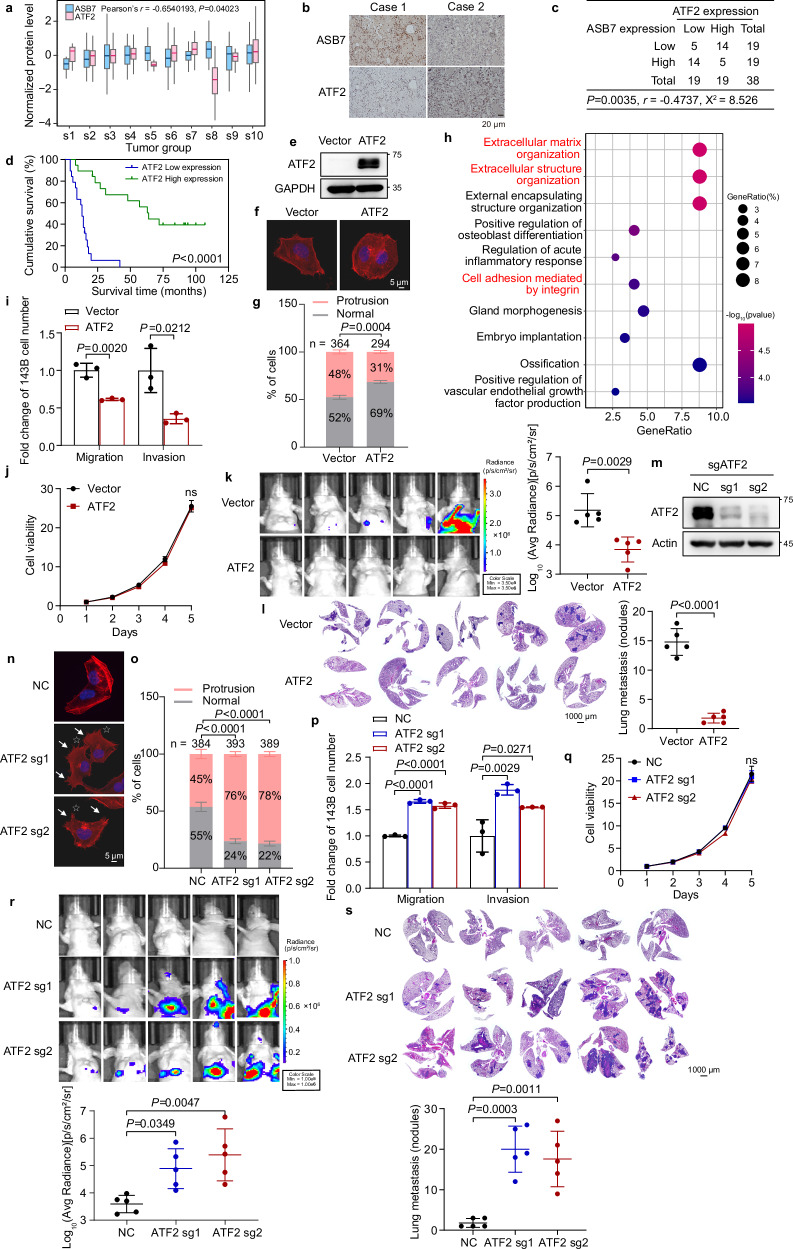
ATF2 inhibits osteosarcoma cell invasion, migration, and lung metastasis. **a** The normalized protein levels of ASB7 and ATF2 across unbiased re-classified tumor subtypes were plotted using data from the UALCAN database^14,36^. Pearson’s correlation coefficient (*r*) was calculated to assess the relationship between ASB7 and ATF2 protein levels across tumor subtypes. **b** Representative images of immunohistochemical staining of ASB7 and ATF2 in osteosarcoma tissues. **c** A contingency table was constructed to display the stratification of 38 osteosarcoma cases based on high or low expression of ASB7 and ATF2, in which the dichotomization was determined by the median IHC score obtained from the HALO system. The Spearman correlation coefficient was used to quantify the association, while Pearson’s chi-square test was used to determine the corresponding *χ*^2^ statistic and *P* value. **d** Kaplan-Meier survival analysis of osteosarcoma patients based on ATF2 protein expression level (low, *n* = 19; high, *n* = 19). The *P* value from the log-rank test is shown, with the high and low-expression groups dichotomized by the median IHC score obtained from the HALO system. **e**–**j** 143B cells expressing vector or ATF2 were subjected to western blotting (**e**), Actin-Tracker staining (**f**), quantification of cells exhibiting finger-like or fan-/wave-like protrusions (**g**), cell migration and invasion assays (*n* = 3 independent experiments) (**i**), and MTT assays (*n* = 3 independent experiments) (**j**). **h** GO analysis of genes upregulated in 143B cells in which ATF2 was knocked down by siRNAs. **k**, **l** In vivo fluorescence imaging and the corresponding quantitative analysis (**k**), H&E staining and the corresponding quantitative analysis (**l**) of mice bearing orthotopic 143B cells expressing luciferase together with either vector or ATF2 (*n* = 5 mice per group). **m**–**q** In 143B cells expressing sgRNAs targeting either non-target control or ATF2, western blotting (**m**), Actin-Tracker staining (**n**), quantification of cells with finger-like or fan-/wave-like protrusions (**o**), cell migration and invasion assays (*n* = 3 independent experiments) (**p**), and MTT assays (*n* = 3 independent experiments) (**q**) were performed. **r,** **s** In mice bearing orthotopic 143B cells expressing luciferase together with sgRNAs targeting non-target control or ATF2, in vivo fluorescence imaging (**r**), H&E staining (**s**) of lung sections and the corresponding quantitative analyses were performed (*n* = 5 mice per group). Data are shown as the mean ± S.D. *P* values were derived from unpaired two-tailed Student’s *t*-test for the data in **g**, **i**, **k**, **l**, **o**, **p**, **r**, **s**; from two-way ANOVA followed by Sidak’s test for the data in **j**; or from two-way ANOVA followed by Tukey’s test for the data in **q**.

Our previous mass spectrometry study revealed a set of potential ASB7 targets, including the reported substrate PSRC1 (DDA3), supporting the reliability of our experimental approach^10^. Notably, activating transcription factor 2 (ATF2) was among the most downregulated proteins^11^. Consistently, CPTAC data showed a negative correlation (Pearson’s *r* = –0.65; *P* = 0.04) between ASB7 and ATF2 protein levels across multiple tumor subtypes^14^ (Fig. 2a). ATF2, a ubiquitously expressed transcription factor, plays pivotal roles in diverse biological processes and pathological conditions^15,16^. In colorectal cancer, the loss of ATF2 expression promotes tumor invasion through the upregulation of the expression of the oncogene TROP2^17^, demonstrating the inhibitory effect of ATF2 on tumor cell migration. Therefore, we further investigated the regulatory relationship between ASB7 and ATF2 and examined whether ATF2 is involved in osteosarcoma metastasis.

Immunohistochemistry (IHC) analysis of osteosarcoma samples revealed that the protein expression of ASB7 was inversely correlated with that of ATF2 (Fig. 2b, c). Low ATF2 expression was associated with poor prognosis (Fig. 2d), suggesting that ASB7 and ATF2 play opposing roles in osteosarcoma progression. To further evaluate the function of ATF2, we generated 143B cells with ATF2 overexpression (Fig. 2e) and observed decreased protrusion formation in 143B cells (Fig. 2f, g). RNA-seq analysis revealed that upregulated genes were enriched in cell adhesion-related processes in ATF2-knockdown 143B cells (Fig. 2h), indicating that in contrast to ASB7, ATF2 might suppress tumor migration. Moreover, ATF2 overexpression inhibited cell migration and invasion but had minimal effects on cell viability in 143B cells (Fig. 2i, j). Additionally, lung metastases in mice with orthotopic xenografts decreased when ATF2 was overexpressed (Fig. 2k, l). In contrast, ATF2 knockout promoted cell protrusion formation, migration, invasion, and lung metastasis but led to minimal alterations in cell viability (Fig. 2m–s). These results suggest that ATF2 is negatively correlated with ASB7 at the protein level and that ATF2 suppresses osteosarcoma migration, invasion and lung metastasis.

### CUL5^ASB7^ ubiquitinates ATF2 at K383 for proteasomal degradation

**Fig. 3:**
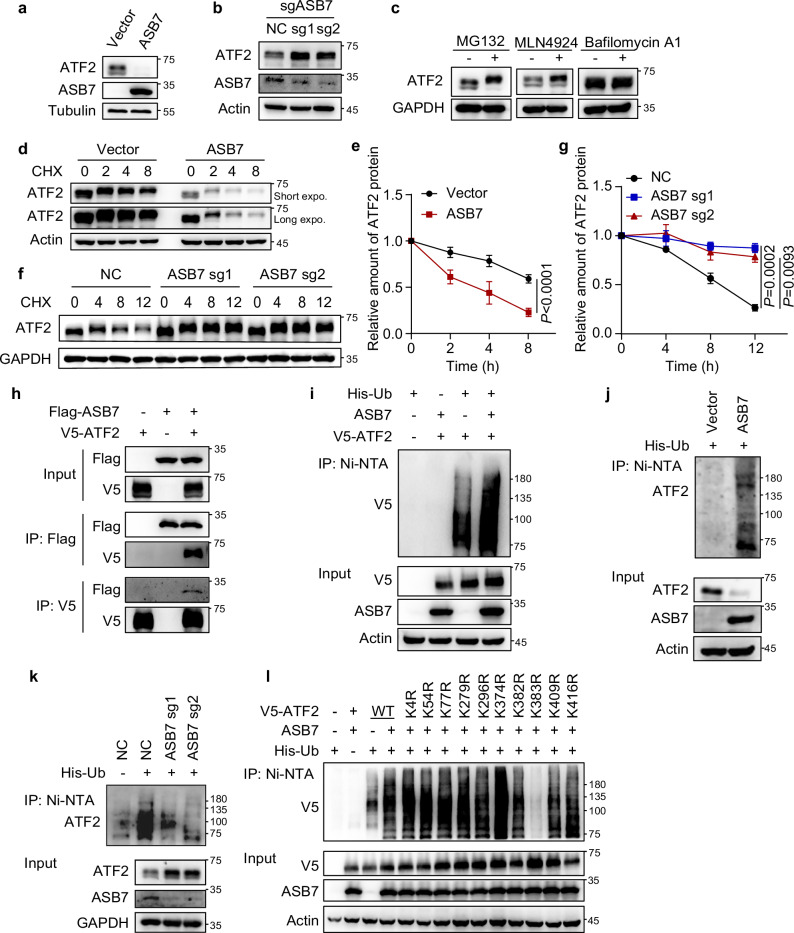
CUL5^ASB7^ promotes the proteasomal degradation of ATF2 through K383 ubiquitination. **a**, **b** ATF2 protein levels were analyzed by western blotting in 143B cells with ASB7 overexpression (**a**) or knockout (**b**). **c** Western blotting analysis of ATF2 protein levels in 143B cells treated with MG132 (10 μM, 6 h), MLN4924 (1 μM, 12 h), or bafilomycin A1 (200 nM, 6 h). **d**–**g** Western blotting analysis of ATF2 protein levels at the indicated times following CHX (40 μg/mL) treatment in 143B cells with ASB7 overexpression (**d**) or knockout (**f**). Panels **e** and **g** show the corresponding quantitative data from these experiments. *n* = 3 independent experiments. **h** HEK293T cells were co-transfected with the indicated constructs for 24 h and treated with 10 μM MG132 for another 6 h. After that, the cells were lysed and subjected to immunoprecipitation using anti-Flag or anti-V5 beads. **i** HEK293T cells were co-transfected with the indicated constructs for 24 h and subsequently treated with MG132 (10 μM, 6 h). After that, the cells were subjected to a Ni-NTA pull-down assay to examine the exogenous ubiquitination of ATF2. **j**, **k** Endogenous ubiquitination of ATF2 was evaluated by Ni-NTA pull-down assay in 143B cells with ASB7 overexpression (**j**) or knockout (**k**) following transfection with His-Ub and treatment with 10 μM MG132 for 6 h. **l** Ubiquitination analysis of ATF2 mutants. HEK293T cells were co-transfected with the indicated constructs, treated with MG132 (10 μM, 6 h), and subjected to a Ni-NTA pull-down assay to assess ATF2 ubiquitination levels. Data are shown as the mean ± S.D. *P* values were derived from two-way ANOVA followed by Sidak’s multiple comparisons test, as shown in (**e**), or two-way ANOVA followed by Tukey’s multiple comparisons test, as shown in **g**.

To further elucidate the relationship between ASB7 and ATF2, we generated 143B cell lines with either ASB7 overexpression or knockout. ATF2 protein levels but not mRNA levels decreased in these cells, which is consistent with the IHC results for patient samples (Fig. 3a, b; Supplementary Fig. S1a, b). Given that ASB7 complexes with CUL5 to form the CUL5-RING E3 ubiquitin ligase, we hypothesized that ASB7 might directly ubiquitinate ATF2. Indeed, CUL5 knockout cells exhibited elevated ATF2 protein (Supplementary Fig. S1c). Cells treated with the proteasome inhibitor MG132 or the NEDD8-activating enzyme inhibitor MLN4924, which inhibits the activation of CUL5, both increased ATF2 protein levels, while the autophagy-lysosome inhibitor bafilomycin A1 had a minimal effect (Fig. 3c). These data indicate that ATF2 can be degraded through the CUL5-dependent ubiquitin-proteasome pathway.

Furthermore, inhibition of the translation of ATF2 using cycloheximide (CHX) revealed that ASB7 overexpression shortened the half-life of the ATF2 protein, whereas ASB7 knockout increased it (Fig. 3d–g), indicating that ASB7 regulates ATF2 protein stability. Moreover, co-IP assays demonstrated a direct interaction between ASB7 and ATF2 (Fig. 3h). Co-IP assays using ASB7 and ATF2 truncation mutants revealed that the interaction between the N-terminal region containing the ANK domain of ASB7 and the N-terminal region containing the ZnF domain of ATF2 mediated their interaction (Supplementary Fig. S1d, e). ASB7 overexpression promoted the ubiquitination of both exogenous and endogenous ATF2 (Fig. 3i, j), whereas ASB7-knockout reduced endogenous ATF2 ubiquitination (Fig. 3k), suggesting that ASB7 might directly ubiquitinate ATF2. Finally, using mass spectrometry and point mutation assays, we revealed that ASB7 ubiquitinates ATF2 at K383 (Fig. 3l; Supplementary Fig. S1f, g). These findings establish that CUL5^ASB7^ ubiquitinates ATF2 at K383 for proteasomal degradation.

### ASB7 promotes tumor lung metastasis by inhibiting ATF2

**Fig. 4:**
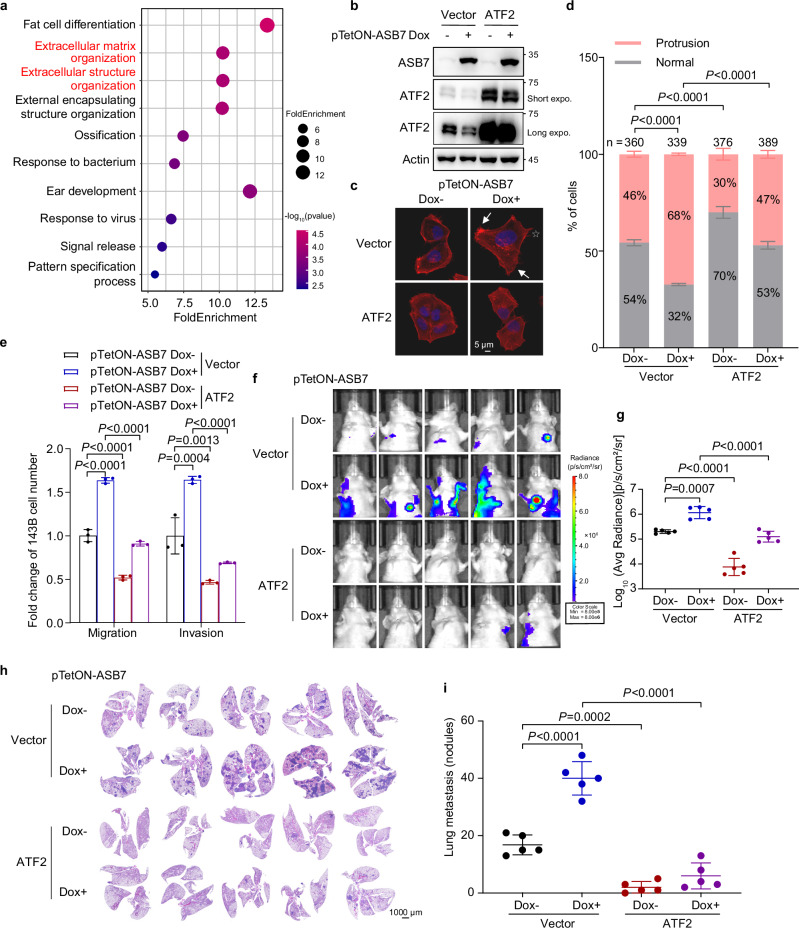
ASB7 facilitates tumor lung metastasis through the inhibition of ATF2. **a** GO analysis of genes upregulated in both 143B cells with ASB7 overexpression and those with ATF2 knockdown by siRNAs. **b**–**e** 143B cells expressing doxycycline-induced ASB7 together with either vector or ATF2 were subjected to western blotting (**b**), Actin-Tracker staining (**c**), quantification of cells with finger-like or fan-/wave-like protrusions (**d**), and migration and invasion assays (**e**). *n* = 3 independent experiments. **f**–**i** In mice bearing orthotopic 143B-Luc cells expressing doxycycline-induced ASB7 together with either vector or ATF2, in vivo lung fluorescence images (**f**) and H&E staining of lung sections (**h**) are shown, along with the corresponding quantitative analyses in **g**, **i**, respectively. *n* = 5 mice per group. Data are shown as the mean ± S.D. Statistical significance in **d**, **e**, **g**, **i** was assessed by an unpaired two-tailed Student’s *t*-test.

To elucidate the effect of ASB7-mediated ATF2 degradation in osteosarcoma, we first analyzed genes whose expression was upregulated in both ASB7-overexpressing cells and ATF2-knockdown cells. Gene Ontology (GO) analysis revealed enrichment in the extracellular matrix organization process (Fig. 4a), suggesting that ASB7 promotes tumor metastasis by facilitating the degradation of ATF2. Reintroduction of ATF2 into doxycycline-induced ASB7-overexpressing 143B cells attenuated cell protrusion formation, migration and invasion (Fig. 4b–e). In mice with orthotopic xenografts, ATF2 overexpression reversed osteosarcoma lung metastasis induced by ASB7 upregulation (Fig. 4f–i). These results suggest that ASB7 promotes osteosarcoma lung metastasis by mediating ATF2 degradation.

### ATF2 suppresses ITGB2 transcription and inhibits tumor lung metastasis

**Fig. 5:**
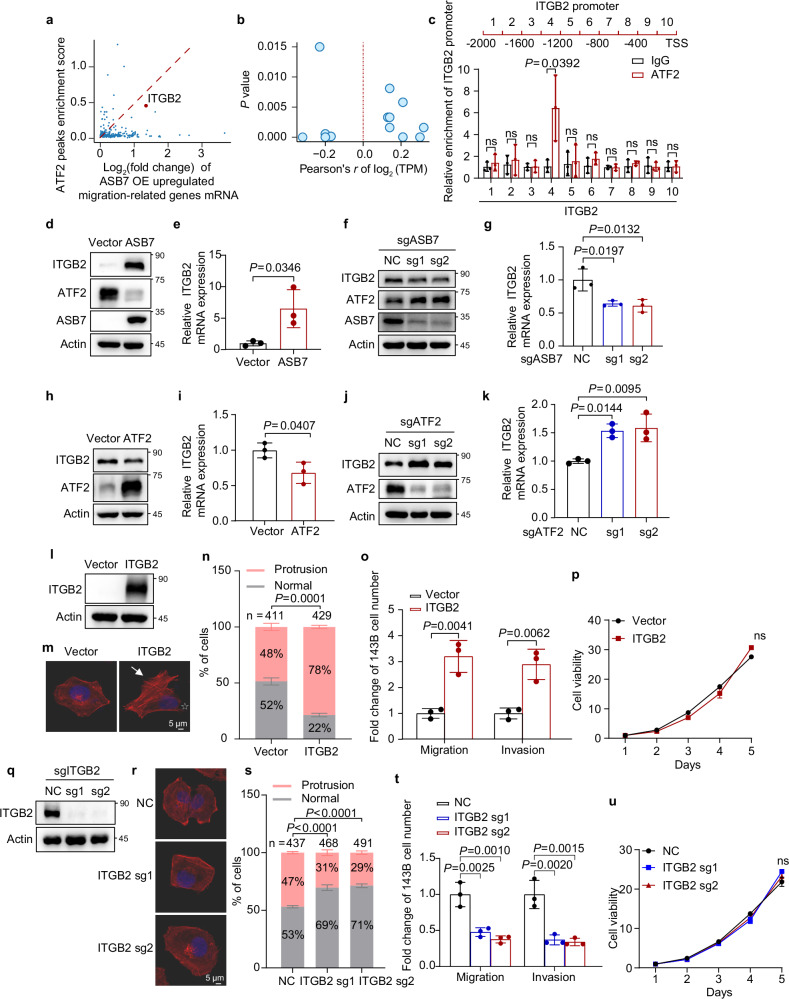
ATF2 suppresses ITGB2 transcription and inhibits tumor lung metastasis. **a** The ATF2 peak enrichment scores and expression fold changes for migration-related genes upregulated upon ASB7 overexpression were plotted. The dashed red line indicates the ideal distribution of genes that are both strongly bound by ATF2 and upregulated upon ASB7 overexpression. **b** Pearson’s correlation coefficients (*r*) and the corresponding *P* values of ATF2 and ITGB2 expression across tumor types were plotted using TCGA transcriptomic data from the GEPIA database. **c** Schematic of the ITGB2 promoter region (top) and the subsequent ChIP-qPCR assay used to identify the site enriched in ATF2 in 143B cells. **d**–**k** ITGB2 protein levels were analyzed by western blotting in 143B cells with ASB7 overexpression (**d**), ASB7 knockout (**f**), ATF2 overexpression (**h**), and ATF2 knockout (**j**), respectively. ITGB2 mRNA levels were analyzed by qRT-PCR in 143B cells with ASB7 overexpression (**e**), ASB7 knockout (**g**), ATF2 overexpression (**i**), and ATF2 knockout (**k**), respectively. **l**–**p** 143B cells expressing vector or ITGB2 were analyzed using western blotting (**l**) and Actin-Tracker staining (**m**). The proportion of cells with finger-like or fan-/wave-like protrusion morphology was quantified (**n**). Additionally, cell migration and invasion assays (**o**) and MTT assays (**p**) were performed. **q**–**u** 143B cells expressing sgRNAs targeting either non-target control or ITGB2 were subjected to western blotting (**q**), Actin-Tracker staining (**r**), quantification of cells with finger-like or fan-/wave-like protrusions (**s**), cell migration and invasion assays (**t**), and MTT assays (**u**). The data are shown as the mean ± S.D. from three independent experiments. *P* values were derived from unpaired two-tailed Student’s *t*-test for the data in **c**, **e**, **g**, **i**, **k**, **n**, **o**, **s**, **t** and from two-way ANOVA followed by either Sidak’s or Tukey’s multiple comparisons tests for the data in **p**, **u**, respectively.

To identify key downstream effectors of ASB7-mediated ATF2 degradation in promoting lung metastasis, we performed an integrative analysis combining RNA-seq data from ASB7-overexpressing cells with ATF2 ChIP-seq data from the ENCODE database. Interestingly, ITGB2, an integrin beta chain, stood out as both one of the most upregulated genes upon ASB7 overexpression and one of the strongest ATF2-bound targets at the promoter among the migration-related genes (Fig. 5a), suggesting that ITGB2 is co-regulated by ASB7 and ATF2. Consistent with these findings, the TCGA transcriptomic data revealed a significant correlation between the expression of ATF2 and ITGB2 across multiple tumors, with sarcoma showing the strongest negative correlation (Fig. 5b), suggesting that ITGB2 is a transcriptional target of ATF2. ChIP-qPCR further confirmed the direct binding of ATF2 to the ITGB2 promoter in 143B cells (Fig. 5c). The overexpression of ASB7 substantially increased the ITGB2 mRNA and protein levels, whereas the knockout of ASB7 had the opposite effect (Fig. 5d–g). Conversely, overexpression of ATF2 decreased ITGB2 expression, whereas knockout of ATF2 increased its expression (Fig. 5h–k). These findings indicate that ATF2 suppresses ITGB2 transcription through direct binding to its promoter and that ASB7-mediated degradation of ATF2 promotes ITGB2 transcription.

Given that integrins serve as primary molecular links between cells and the extracellular matrix (ECM) while also mediating cell-cell interactions^18^ and play key roles in tumor metastasis^19,20^, we hypothesized that ITGB2 is an important downstream effector of osteosarcoma metastasis. To test this hypothesis, we used 143B cells with either ITGB2 overexpression or knockout. Indeed, ITGB2 overexpression substantially promoted cell protrusion formation, migration and invasion but had a minimal effect on cell viability (Fig. 5l–p). In contrast, ITGB2 knockout decreased cell protrusion formation, migration and invasion but did not significantly affect cell viability (Fig. 5q–u). In vivo, mice with orthotopic 143B osteosarcoma showed increased lung metastases upon ITGB2 overexpression (Supplementary Fig. S2a–d). Conversely, ITGB2 knockout led to a reduction in the lung metastatic burden (Supplementary Fig. S2e, f). Together, these data indicate that ASB7-mediated ATF2 degradation leads to the transcriptional de-repression of ITGB2. The upregulated ITGB2 together with other downstream effectors of ASB7-ATF2 promotes tumor metastasis.

### ATF2 recruits HDAC6 to suppress ITGB2 transcription

**Fig. 6:**
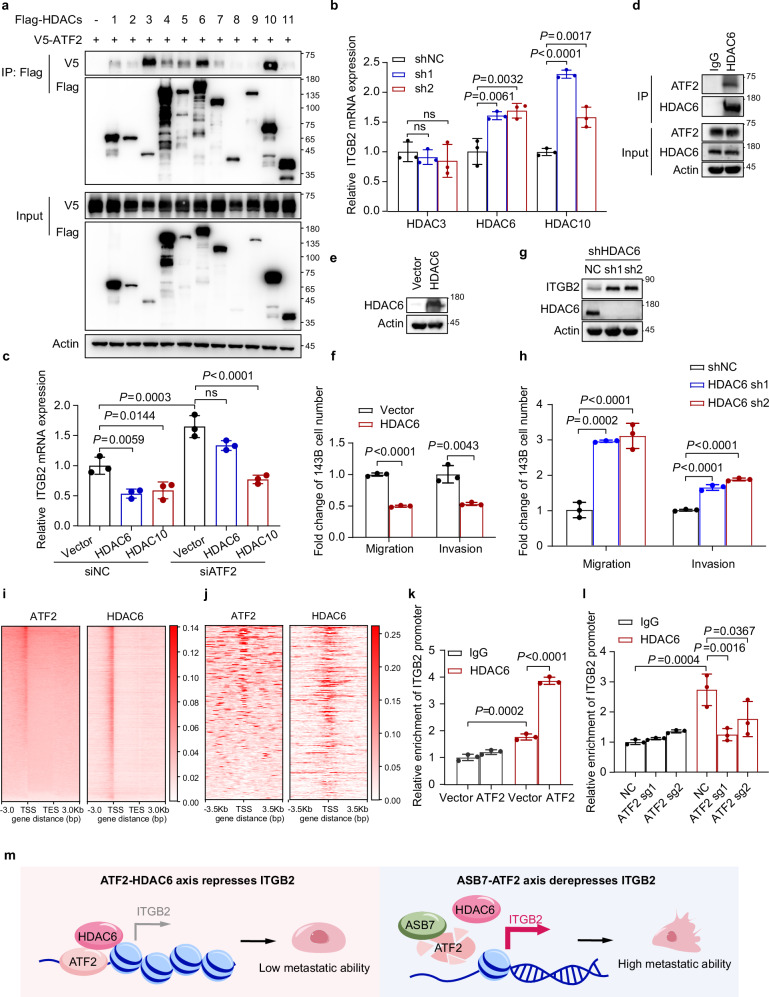
ATF2 suppresses ITGB2 transcription by recruiting HDAC6. **a** HEK293T cells were co-transfected with V5-ATF2 and Flag-HDACs, and the interaction between ATF2 and HDACs was assessed by immunoprecipitation with anti-Flag beads. **b** qRT-PCR analysis of ITGB2 mRNA levels in 143B cells after knockdown of HDAC3, HDAC6, or HDAC10. **c** qRT-PCR analysis of ITGB2 mRNA levels in 143B cells with the indicated constructs. **d** Co-immunoprecipitation analysis of the endogenous interaction between HDAC6 and ATF2 in 143B cells. **e**, **f** 143B cells stably expressing vector or HDAC6 were analyzed by western blotting (**e**) and cell migration and invasion assays (*n* = 3 independent experiments) (**f**). **g**, **h** 143B cells expressing shRNAs targeting either non-target control or HDAC6 were subjected to western blotting (**g**) and cell migration and invasion assays (*n* = 3 independent experiments) (**h**). **i** Heatmaps showing the genome-wide distributions of ATF2 ChIP-seq signals (ENCODE) and HDAC6 CUT&Tag signals across gene bodies with ± 3.0 kb flanking regions. **j** Heatmaps showing the distributions of ATF2 ChIP-seq signals (ENCODE) and HDAC6 CUT&Tag signals centered on the TSSs of ATF2-bound promoters associated with migration-related genes. The signals are plotted ± 3.5 kb around the TSS. **k**, **l** ChIP-qPCR analysis of HDAC6 enrichment at the ITGB2 promoter in 143B cells with ATF2 overexpression (**k**) or knockout (**l**). **m** Model of the ASB7-ATF2/HDAC6-ITGB2 axis in osteosarcoma. ATF2 recruits HDAC6 to transcriptionally repress ITGB2 and inhibit cell movement, while ASB7-mediated ATF2 degradation relieves this repression, activating ITGB2 expression and inducing protrusion formation to drive metastasis. The data are shown as the mean ± S.D. from three independent experiments. *P* values were derived from unpaired two-tailed Student’s *t*-test, as shown in **b**, **c**, **f**, **h**, **k**, **l**.

We next sought to determine how ATF2 suppresses ITGB2 transcription through promoter binding. Studies have demonstrated that ATF2 can recruit HDAC3 through a non-canonical mechanism to regulate inflammatory gene expression^21^. Given that histone deacetylases (HDACs) can inhibit transcription via deacetylation, we tested whether ATF2 recruits HDACs to inhibit ITGB2 transcription^22^. To this end, we first identified the HDACs that interact with ATF2. Co-transfection and co-IP assays revealed that ATF2 interacts with HDAC3, HDAC6, and HDAC10 (Fig. 6a). However, knockdown of either HDAC6 or HDAC10, but not HDAC3, by shRNAs increased ITGB2 mRNA expression (Fig. 6b). Next, we assessed HDAC6 and HDAC10 activity under ATF2-deficient conditions. In cells with non-target control siRNAs, overexpression of either HDAC6 or HDAC10 reduced ITGB2 expression, whereas in cells with ATF2-targeted siRNAs, only HDAC10 overexpression retained the transcriptional suppression of ITGB2 (Fig. 6c). These results demonstrate that both HDAC6 and HDAC10 can inhibit ITGB2 transcription but that only HDAC6 requires ATF2. Indeed, endogenous IP assays revealed that HDAC6 interacts with ATF2 (Fig. 6d). Furthermore, we constructed HDAC6 overexpression and knockdown cell lines and observed that HDAC6 overexpression inhibited cell migration, whereas knockdown promoted it (Fig. 6e–h). Additionally, analysis of ATF2 ChIP-seq data from ENCODE and HDAC6 CUT&Tag data revealed widespread co-occupancy of ATF2 and HDAC6 at genome-wide gene promoters, including promoters of genes implicated in cell migration (Fig. 6i, j). Furthermore, ChIP-qPCR confirmed the binding of HDAC6 to the ITGB2 promoter. ATF2 overexpression increased HDAC6 occupancy, whereas ATF2 knockout reduced it (Fig. 6k, l). These data established that ATF2 recruits HDAC6 to the promoter of ITGB2, leading to the suppression of ITGB2 transcription.

## Discussion

Osteosarcoma has a high potential for lung metastasis, highlighting the importance of further exploration of its metastatic mechanisms. In this study, we demonstrated that ASB7 upregulation or ATF2 downregulation promotes osteosarcoma metastasis in cells and in mouse orthotopic xenograft models. In tumor tissues, high expression of ASB7 or low expression of ATF2 was correlated with a poor prognosis. Furthermore, we revealed that ATF2 binds to the promoters of ITGB2 and other tumor migration-related genes and subsequently recruits HDAC6 to suppress the transcription of these genes. ASB7 ubiquitinates ATF2 at K383, leading to its proteasomal degradation. In ASB7-upregulated osteosarcoma, the protein level of ATF2 is decreased. As a result, the transcription of ITGB2 and other tumor migration-related genes is activated, promoting cell protrusion formation, migration, and invasion and ultimately tumor lung metastasis (Fig. 6m).

Frequent amplification and upregulation of ASB7 are observed across multiple tumors, especially in sarcoma and osteosarcoma. However, its role in tumor progression remains poorly understood. In this study, we demonstrate the metastasis-promoting effect of ASB7 upregulation in osteosarcoma. Given that our previous study revealed that ASB7 overexpression leads to decreased SUV39H1 expression and impaired homologous recombination repair, thereby sensitizing tumor cells to PARP inhibitors^11^, it is worth further investigating whether PARP inhibitors could suppress the metastasis of osteosarcoma with upregulated ASB7 expression. Currently, early-phase clinical trials evaluating PARP inhibitors in osteosarcoma and other bone and soft tissue tumors are actively ongoing, as a subset of patients exhibit BRCA1- or BRCA2-deficient-like properties^23,24^. This study may offer potential guidance for more precise patient stratification.

The transcriptional activation function of ATF2 is well established and is mediated through the formation of either homodimers or heterodimers with other transcription factors^25^. A previous study revealed that ATF2 can also activate downstream transcription in LPS-treated macrophages through the recruitment of HDAC3^26^. However, how ATF2 suppresses transcription remains unclear. We revealed that ATF2 recruits HDAC6 to the promoter of *ITGB2* and suppresses its transcription. Given the colocalization of ATF2 and HDAC6 at the promoters of genes related to migration, as revealed by the public datasets for ATF2 and HDAC6, we propose that ATF2 recruits HDAC6 to suppress the transcription of cell migration-related genes.

In summary, this study reveals the regulatory mechanism through which the ASB7-ATF2/HDAC6-ITGB2 axis promotes osteosarcoma lung metastasis, providing potential targets for the development of novel therapeutic strategies against osteosarcoma.

## Materials and methods

### Cell culture, stable transfection, and transient transfection

All the cell lines used in this study were obtained from authenticated sources and maintained under standard conditions. The human cell lines 143B and HEK293T were purchased from ATCC. The luciferase-expressing 143B cell line (143B-Luc) was cultured according to established protocols^27,28^. All the cell lines were grown in DMEM supplemented with 10% FBS and 1% penicillin-streptomycin at 37 °C in a 5% CO₂ atmosphere. Cell line authentication was conducted using short tandem repeat (STR) profiling within six months prior to experimental use, with routine subcultures limited to a maximum of two months to ensure genetic stability and phenotypic consistency.

For stable gene expression, cell lines were engineered to express short hairpin RNAs (shRNAs), single-guide RNAs (sgRNAs), or cDNA constructs through puromycin selection at 0.5 μg/mL or blasticidin S HCl at 5 μg/mL. Transient transfection experiments were performed using optimized protocols for each cell type. HEK293T cells were transfected with plasmids using linear polyethylenimine, while osteosarcoma cells were transfected using Lipofectamine 3000 reagent. For RNA interference experiments, 143B cells were transfected with small interfering RNAs (siRNAs) using Lipofectamine RNAiMAX. Following transfection, the culture medium was replaced with fresh complete medium after 6–8 h to ensure optimal cell viability and transfection efficiency. All oligonucleotide sequences, including siRNAs, shRNAs, and sgRNAs, are provided in Supplementary Tables S1–S3.

### Plasmid construction and lentivirus production

For protein expression studies, ASB7 constructs were generated in pSIN or pTetON vectors, including full-length and truncated versions with optional Flag, SFB, or HA tags. Similarly, ATF2 constructs (wild-type and K-to-R mutants) with optional 3 × Myc or V5 tags were cloned into the pSIN vector. ITGB2 cDNA was amplified and subsequently inserted into the pSIN vector. The Flag-tagged HDAC constructs were obtained from a previous study and are maintained in our laboratory^29^. For gene knockdown experiments, shRNAs targeting HDAC3/6/10 were designed and cloned into the pLKO.1 vector. Additionally, sgRNAs targeting ATF2, ITGB2 or CUL5 were constructed using the lentiCRISPR V2 vector. The sgRNA and shRNA sequences are provided in the Supplementary Tables S1, S2.

### Western blotting and co-IP

Cells were lysed in either RIPA buffer (50 mM Tris-HCl (pH 8.0), 150 mM NaCl, 5 mM EDTA, 0.5% CA-630) or RIPA-SDS lysis buffer (50 mM Tris-HCl (pH 7.5), 150 mM NaCl, 0.125% SDS, 0.125% sodium deoxycholate, 1% Triton X-100) supplemented with protease and phosphatase inhibitors (Calbiochem). For western blotting, the lysates were centrifuged at 12,000× *g* and 4 °C for 15 min, after which the supernatants were denatured at 100 °C for 10 min. For the co-IP assay, the cells were lysed in RIPA-SDS buffer, sonicated, and centrifuged, after which 40 μL of the supernatant was reserved for input analysis. The remaining lysate was incubated for 2 h at 4 °C with anti-Flag agarose (Proteintech), anti-V5 agarose (Proteintech), anti-Myc agarose (Proteintech), streptavidin beads (Cytiva), or Ni-NTA beads (Beyotime).

Proteins were separated by SDS-PAGE, transferred to 0.45 μm PVDF membranes (Millipore), and blocked with 5% nonfat milk for 1 h at room temperature. Blots were probed with antibodies against the following: Flag (CST, #14793), HA (CST, #3724), V5 (CST, #13202), Myc (CST, #2278), ATF2 (CST, #35031), ITGB2 (CST, #73663), HDAC6 (ThermoFisher, PA1-41056), GAPDH (ABclonal, A19056), β-Tubulin (ABclonal, A12289), β-Actin (ABclonal, AC026), HSP70 (Santa Cruz, sc-32239), and CUL5 (Sigma‒Aldrich, HPA002185). Anti-ASB7 antibodies were generated by immunizing rabbits with a recombinant ASB7 fragment (amino acids 100-318; Shanghai Genomics Technology) as previously described^11^.

### RNA extraction and real-time qPCR

Total RNA was isolated using the TIANGEN RNA purification kit, followed by cDNA synthesis with HiScript II Q RT SuperMix for qPCR (Vazyme). Real-time qPCR was performed on a LightCycler 480 II system (Roche) using ChamQ Universal SYBR qPCR Master Mix (Vazyme). Transcript levels were normalized to those of GAPDH or Actin, and relative mRNA levels were calculated using the ΔΔCt method. The reaction specificity was confirmed by melting curve analysis. All primer sequences are provided in Supplementary Table S4.

### RNA sequencing

Total RNA was isolated from 143B cells stably expressing ASB7 or transfected with ATF2 siRNA using TRIzol reagent (Life Technologies, #15596026). RNA sequencing was performed by Novogene (Beijing, China) on an Illumina NovaSeq 6000 platform. Reads were aligned to GRCh38 and analyzed using the nf-core/rnaseq pipeline^30^. Differential gene expression analysis was performed using the DESeq2 package (v1.38.3). Gene Ontology enrichment was performed using the ClusterProfiler package^31^.

### Detection of the ubiquitination site of ATF2 by mass spectrometry

HEK293T cells were co-transfected with ASB7, SFB-ATF2, and HA-ubiquitin plasmids. After 36 h of transfection, the cells were treated with 10 μM MG132 (a proteasome inhibitor) for 6 h to stabilize ubiquitinated proteins. Cells were then lysed in RIPA-SDS buffer supplemented with protease inhibitors and sonicated to fragment genomic DNA. The lysates were clarified by centrifugation at 14,000 × *g* for 20 min at 4 °C. For affinity purification, the supernatants were incubated with streptavidin beads for 4 h at 4 °C. After five washes with RIPA-SDS buffer, the bound proteins were eluted by boiling at 95 °C for 10 min. The eluates were separated by 8% SDS-PAGE, and the band corresponding to SFB-ATF2 was excised for mass spectrometry analysis of ubiquitination sites.

### Ubiquitination assay

Upon reaching 70% confluency, the HEK293T cells were co-transfected with plasmids encoding ASB7, V5-ATF2, and His-ubiquitin, while the ASB7-overexpressing and ASB7-knockout cells were transfected with the His-ubiquitin plasmid alone. After 36 h of transfection, the cells were treated with 10 μM MG132 for 6 h to stabilize ubiquitinated proteins before being lysed in denaturing buffer A (6 M guanidine-HCl, 0.1 M Na₂HPO₄/NaH₂PO₄, 10 mM imidazole, pH 8.0). The lysates were sonicated and centrifuged, and the supernatants were incubated with Ni-NTA beads (Beyotime) for 3 h at room temperature with rotation. The beads were subsequently washed twice with buffer A, twice with buffer A/TI (1:3), and once with buffer TI (25 mM Tris-HCl, 20 mM imidazole, pH 6.8). His-ubiquitinated proteins were eluted by boiling in 1 × SDS loading buffer for 10 min at 100 °C, resolved by SDS-PAGE, and transferred to PVDF membranes for immunoblotting with target antibodies.

### ChIP-qPCR assays

Approximately 2 × 10⁷ cells were cross-linked with 1% formaldehyde for 10 min at room temperature, quenched with 0.125 M glycine for 5 min, and lysed in 1 mL of cell lysis buffer (20 mM Tris-HCl (pH 8.0), 85 mM KCl, 0.5% NP-40) for 30 min at 4 °C. Nuclei were collected by centrifugation at 1000 × *g* for 5 min at 4 °C, and nuclear lysis was performed in 1 mL of nuclear lysis buffer (50 mM Tris-HCl (pH 8.0), 10 mM EDTA, and 1% SDS) for 30 min at room temperature. Chromatin was then sheared into 200–400 bp fragments using a Covaris E220 focused ultrasonicator. The sheared chromatin supernatant was obtained by centrifugation at 12,000 × *g* for 10 min at 4 °C, and 100 μL aliquots were diluted to 1 mL in ChIP Dilution Buffer (0.01% SDS, 1.1% Triton X-100, 1.2 mM EDTA, 16.7 mM Tris-HCl (pH 8.0), and 167 mM NaCl). The samples were immunoprecipitated overnight at 4 °C with anti-ATF2, anti-HDAC6, or control IgG antibodies and then incubated with protein A/G magnetic beads for 5 h at 4 °C. Beads were sequentially washed with low-salt wash buffer (0.1% SDS, 1% Triton X-100, 2 mM EDTA, 20 mM Tris-HCl (pH 8.0), 150 mM NaCl), high-salt wash buffer (0.1% SDS, 1% Triton X-100, 2 mM EDTA, 20 mM Tris-HCl (pH 8.0), 500 mM NaCl), LiCl wash buffer (0.25 M LiCl, 1% NP40, 1% deoxycholate, 1 mM EDTA, 10 mM Tris-HCl (pH 8.0)), and TE buffer (10 mM Tris-HCl (pH 8.0), 1 mM EDTA). Immunoprecipitated DNA was eluted in 100 μL of ChIP Elution Buffer (1% SDS, 0.1 M NaHCO₃) containing RNase A at 37 °C for 30 min, followed by treatment with proteinase K at 65 °C for 2 h and a final incubation at 95 °C for 10 min. DNA was purified using Tiangen’s Universal DNA Purification Kit according to the manufacturer’s protocol and analyzed by quantitative PCR (qPCR). Target sequence enrichment was calculated using the fold-change method relative to control IgG samples.

### Immunofluorescence

Cells at 30% confluence in confocal dishes were fixed with 4% paraformaldehyde for 15 min, permeabilized with 0.5% Triton X-100 for 10 min, and blocked with goat serum for 30 min. F-actin was visualized using Actin-Tracker Red-594 (Beyotime, C2205S) for 1 h, followed by nuclear counterstaining with Hoechst 33342 (Thermo Fisher, H3570) for 3 min. Samples were mounted in antifade mounting medium (Beyotime, P0128M) and either imaged immediately or stored at 4 °C. Cells displaying lamellipodia (fan- or wave-like) or filopodia (needle-like) were considered positive for actin-based protrusions^32^.

### IHC

Paraffin-embedded tissue sections were deparaffinized and dehydrated through a graded series of alcohols. Antigen retrieval was performed using EDTA-Tris buffer (pH 9.0). After blocking with 5% goat serum for 30 min, the sections were incubated overnight at 4 °C with primary antibodies, including anti-ASB7 (1:750; Shanghai Genomics Technology) and anti-ATF2 (1:500; CST 35031). Following PBS washes, endogenous peroxidase activity was blocked with 3% H₂O₂ for 10 min, and the sections were incubated with species-matched secondary antibodies before DAB development (Dako Omnis). ASB7 and ATF2 protein levels were quantified using Halo software. Protein levels were dichotomized into high or low groups based on median IHC scores. The associations between proteins were assessed using the *χ*^2^ test and Pearson correlation.

### MTT assay

Cells were seeded in 96-well plates at a density of 1400 cells per well and cultured for 24 h. After the cells were incubated with 0.5 mg/mL MTT (3-(4,5-dimethylthiazol-2-yl)-2,5-diphenyltetrazolium bromide) for 4 h at 37 °C, the medium was carefully removed and replaced with 100 μL of DMSO per well. The plates were gently agitated for 10 min to completely dissolve the formazan crystals before the optical density was measured at 490 nm for 5 consecutive days using a microplate reader.

### Migration and invasion assays

Cell migration and invasion assays were performed using 24-well Boyden chambers with 8 μm pore inserts. For invasion assays, inserts were pre-coated with Matrigel, while uncoated inserts were used for migration studies. Cells (5 × 10⁴/well) were seeded in serum-free medium in the upper chambers and incubated at 37 °C for 8–10 h. After incubation, the cells were fixed with 4% paraformaldehyde for 10 min, washed with PBS, and stained with crystal violet for visualization and quantification of migrated or invaded cells.

### Animal experiments

BALB/c-nude mice were purchased from Vital River Laboratories (Beijing, China) and Zhuhai BesTest Bio-Tech Co., Ltd. (Zhuhai, China). Four-week-old male BALB/c-nude mice were randomly allocated to experimental groups (*n* = 5 per group). Luciferase-expressing 143B osteosarcoma cells were engineered to generate stable overexpression, knockout, or rescue cell lines, as described in the manuscript. A suspension of 7 × 10⁵ cells in PBS was orthotopically injected into the tibia of each mouse. Tumor progression and metastasis were monitored using an IVIS Lumina Imaging System. Upon confirmation of significant lung metastasis, the mice were euthanized at the experimental endpoint. Lung tissues were harvested and subjected to hematoxylin and eosin (H&E) staining.

### Sequencing data analysis

ATF2 ChIP-seq data were obtained from ENCODE (ENCSR047BUZ). The corresponding input data were obtained from ENCODE (ENCSR121CWV). HDAC6 CUT&Tag data were obtained from GSE253743^33^. ATF2 ChIP-seq data were aligned to GRCh38 and reanalyzed using the nf-core/chipseq pipeline^30^. HDAC6 CUT&Tag data were aligned to GRCh38 and reanalyzed using the nf-core/cutandrun pipeline^30^. The enrichment scores indicating ATF2 occupancy at the promoters of genes related to cell migration were calculated using plotHeatmap in deepTools software^34^. The Pearson’s *r* values of ATF2 and ITGB2 across TCGA tumors were obtained from the GEPIA database^35^.

### Statistical analysis

All data are presented as mean ± standard deviation (SD). Error bars represent SD. Statistical analyses were performed using GraphPad Prism. Comparisons between two groups of migration, invasion, qPCR and mouse lung metastasis analysis assays were conducted using an unpaired two-tailed Student’s *t*-test, and MTT assays were analyzed using two-way ANOVA. Migration, invasion, and qPCR experiments were performed with three independent biological replicates (*n* = 3). Due to the limited sample size, formal testing of normality was not performed. Statistical analyses were conducted assuming approximate normal distribution, as commonly applied in similar experimental designs. A *P* value < 0.05 was considered statistically significant.

## Supplementary information

Supplementary Material

## Data Availability

The datasets generated and/or analyzed in this study are available from the corresponding authors upon reasonable request.
