# Supplementary material for: ASB7 promotes osteosarcoma lung metastasis through ubiquitin-mediated degradation of ATF2
Source: Cell Discov. 2026 May 5;12:31. doi: 10.1038/s41421-026-00890-9 (PMC13144305; doi:10.1038/s41421-026-00890-9)
Supplement: Supplementary file 1 — Supplementary Material [file 41421_2026_890_MOESM1_ESM.pdf]

Supplementary Information for

ASB7 promotes osteosarcoma lung metastasis through ubiquitin-mediated ATF2 degradation

Yezi Zou[1, 2#], Jianliang Zhong[1, 2#], Lanqing Huo[1#], Jiali Chen[1#], Xinhao Yu[1#], Jingxuan Wang[1], Zhenxuan Chen[1], Lifeng Yin[3], Cuiling Zeng[1], Xia Zhang[4], Shan Han[1], Ruhua Zhang[1], Xing-Ding Zhang[2]*, Tiebang Kang[1]*, Liwen Zhou[1]*

*Correspondence to: **Liwen Zhou** (zhoulw@sysucc.org.cn); **Tiebang Kang** (kangtb@sysucc.org.cn); **Xing-Ding Zhang** (zhangxd39@mail.sysu.edu.cn)

The following sections include:

Supplementary Figures S1-S2

Supplementary Table S1-S4

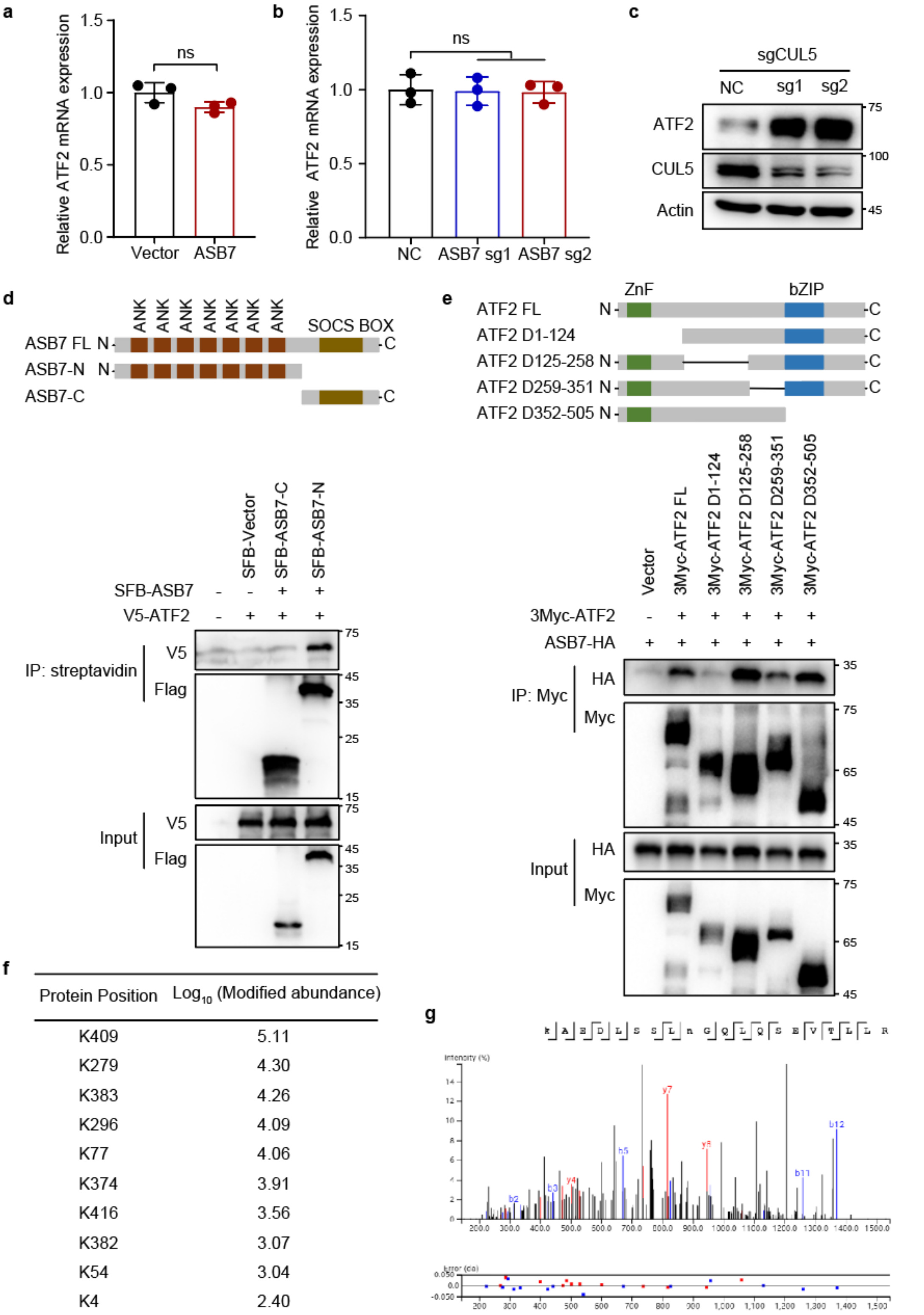

Protein Position	Log_{10} (Modified abundance)
K409	5.11
K279	4.30
K383	4.26
K296	4.09
K77	4.06
K374	3.91
K416	3.56
K382	3.07
K54	3.04
K4	2.40

Supplementary Fig. S1 ASB7-CUL5 E3 ligase complex interacts with and ubiquitinates ATF2 at K383. a, b ATF2 mRNA levels were analyzed by qRT-PCR in 143B cells with ASB7 overexpression (**a**) or knockout (**b**). Data are shown as mean ± SD from three independent experiments. *P* values were derived from two-tailed unpaired Student's *t*-test. **c** Western blot analysis of ATF2 protein levels in CUL5

knockout 143B cells. **d** Schematic of the ASB7 domain and truncation constructs. HEK293T cells were co-transfected with V5-ATF2 and SFB-tagged ASB7 truncations, treated with MG132 (10 μM, 6 h), and subjected to immunoprecipitation with streptavidin beads to identify the domains mediating ASB7 and ATF2 interaction. **e** Schematic of the ATF2 domain and truncation constructs. HEK293T cells were co-transfected with ASB7-HA and 3Myc-tagged ATF2 truncations, treated with MG132 (10 μM, 6 h), and subjected to immunoprecipitation with anti-Myc beads to identify the ATF2 domains interacting with ASB7. **f** HEK293T cells were co-transfected with ASB7, SFB-ATF2, and HA-Ub plasmids. After 36 hours, cells were treated with MG132 (10 μM, 6 h), followed by streptavidin beads pull-down and ubiquitination mass spectrometry analysis. Ubiquitination intensities of identified sites are presented as $\log_{10}$-transformed values in descending order. **g** Mass spectrometry confirming ATF2 ubiquitination at the K383 site.

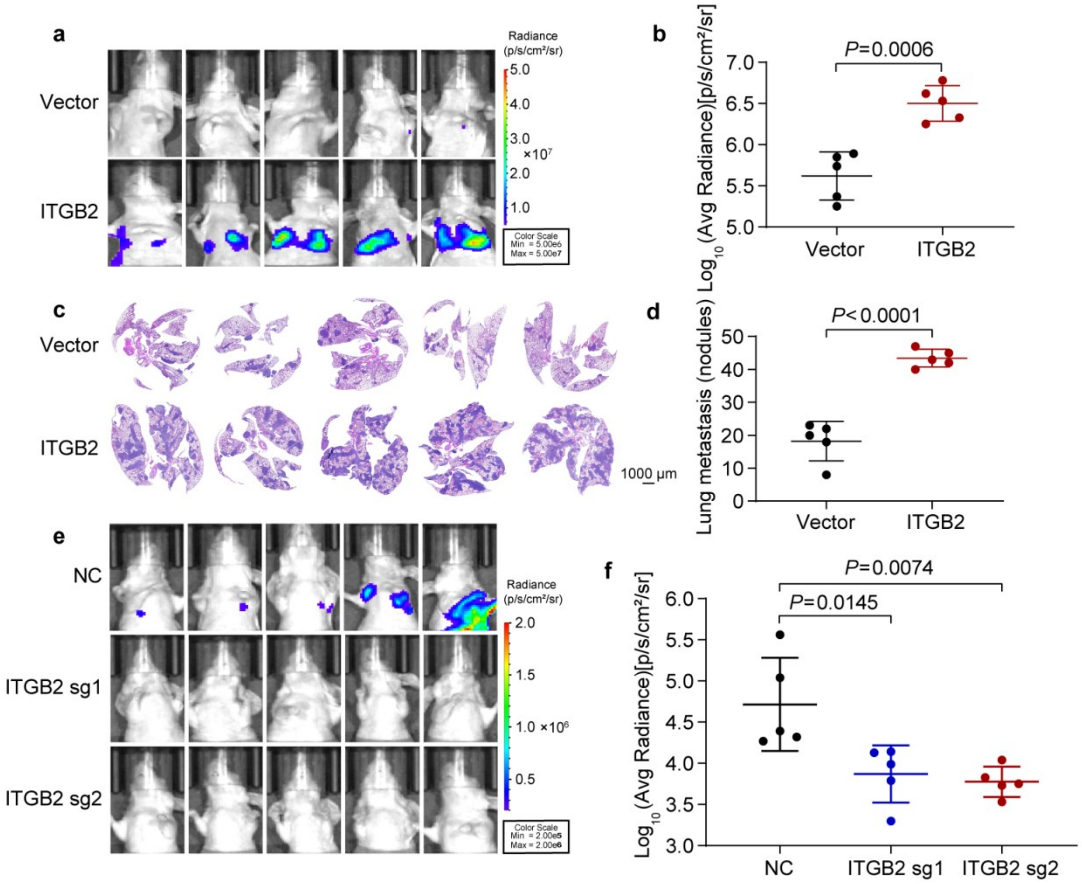

Supplementary Fig. S2 ITGB2 enhances osteosarcoma lung metastasis. a-d Mice implanted with 143B-Luc cells with ITGB2 overexpression were analyzed by bioluminescence imaging of lung (**a**) and H&E staining of lung sections (**c**), with corresponding quantifications shown in (**b**) and (**d**), respectively. **e**, **f** Mice implanted with 143B-Luc cells expressing sgRNAs targeting non-targeting control or ITGB2 were monitored by IVIS imaging (**e**), with the corresponding quantified data shown in (**f**). Data are presented as mean ± SD (n = 5 mice per group), with P values derived from two-tailed unpaired Student's t-test in **b**, **d**, and **f**.

Supplementary Table S1 shRNA Target Sequences

Gene	Target Sequences (5'→3')
HDAC3 #1	AGGCTTCACCAAGAGTCTTAA
HDAC3 #2	GAACAACAAGATCTGTGATAT
HDAC6 #1	TGGTTCACAGCCTAGAATATA
HDAC6 #2	AGACACCTACGACTCAGTTTA
HDAC10 #1	ACAACGCCGGATATCACATTG
HDAC10 #2	GGAAGCTCCTGTACCTCTTAG

Supplementary Table S2 sgRNA Target Sequences

Gene	Target Sequences (5'→3')
ATF2 sg1	CTCAAGTACTGTAATCACCC
ATF2 sg2	TATCATAAGAAGCAAAATTG
ITGB2 sg1	ACGTGCCTGAAGGCAAACGG
ITGB2 sg2	TGCAAGGAGAGGGACTCAGA
CUL5 sg1	AGTAATTGGCAAGCAGCTCA
CUL5 sg2	CAATACAGCGAGCTGAAACG

Supplementary Table S3 Synthetic siRNA Target Sequences

Gene	Target Sequences (5'→3')
siATF2	GCAACACCTATCATAAGAA

Supplementary Table S4 Primers of qPCR

Gene	Sequences (5'→3')
GAPDH-F	TGACTTCAACAGCGACACCC
GAPDH-R	CTGGTGGTCCAGGGGTCTTA
Actin-F	CATGTACGTTGCTATCCAGGC
Actin-R	CTCCTTAATGTCACGCACGAT
ATF2-F	TGTGAATTCTGCCAGGCAAT
ATF2-R	CTCGTTGGTAAAACGCTGGC
ITGB2-F	GACCCAGGGCAGACTGGTA
ITGB2-R	TGCACTCCTGAGAGAGGACG
